# Transcranial alternating current stimulation for the treatment of major depressive disorder: from basic mechanisms toward clinical applications

**DOI:** 10.3389/fnhum.2023.1197393

**Published:** 2023-09-04

**Authors:** Ruibo Pan, Shengfeng Ye, Yun Zhong, Qiaozhen Chen, Ying Cai

**Affiliations:** ^1^Department of Psychiatry, The Second Affiliated Hospital, Zhejiang University School of Medicine, Hangzhou, China; ^2^Department of Psychology and Behavioral Science, Zhejiang University, Hangzhou, China; ^3^Key Laboratory of Medical Molecular Imaging of Zhejiang Province, Hangzhou, China

**Keywords:** transcranial alternating current stimulation (tACS), major depressive disorder (MDD), brain oscillations, functional connectivity, brain networks

## Abstract

Non-pharmacological treatment is essential for patients with major depressive disorder (MDD) that is medication resistant or who are unable to take medications. Transcranial alternating current stimulation (tACS) is a non-invasive brain stimulation method that manipulates neural oscillations. In recent years, tACS has attracted substantial attention for its potential as an MDD treatment. This review summarizes the latest advances in tACS treatment for MDD and outlines future directions for promoting its clinical application. We first introduce the neurophysiological mechanism of tACS and its novel developments. In particular, two well-validated tACS techniques have high application potential: high-definition tACS targeting local brain oscillations and bifocal tACS modulating interarea functional connectivity. Accordingly, we summarize the underlying mechanisms of tACS modulation for MDD. We sort out the local oscillation abnormalities within the reward network and the interarea oscillatory synchronizations among multiple MDD-related networks in MDD patients, which provide potential modulation targets of tACS interventions. Furthermore, we review the latest clinical studies on tACS treatment for MDD, which were based on different modulation mechanisms and reported alleviations in MDD symptoms. Finally, we discuss the main challenges of current tACS treatments for MDD and outline future directions to improve intervention target selection, tACS implementation, and clinical validations.

## Introduction

Major depressive disorder (MDD) is a severe mental disorder with core symptoms of low mood and anhedonia that causes cognitive impairment and increases the risks of comorbidities and suicide (Alexopoulos, 2005; McCarron et al., 2021). The COVID-19 pandemic has greatly exacerbated the disease burden of depressive disorders, with an estimated 53.2 million additional cases of MDD globally (COVID-19 Mental Disorders Collaborators, 2021). The latest data indicate that MDD affects approximately 280 million people, which is 3.8% of the global population (World Health Organization, 2021)^1^ and has become one of the leading causes of disability worldwide (GBD 2019 Mental Disorders Collaborators, 2022). Antidepressant drugs have been the first-line treatment for MDD, but they cannot meet clinical needs in terms of efficacy or side effects (Israel, 2010). Studies have reported that one-third of MDD cases are medication resistant (Trivedi et al., 2006; Zhdanava et al., 2021). Moreover, drug treatment is also highly limited among special populations such as children and adolescents and pregnant or breastfeeding women.

Non-invasive brain stimulation methods, such as electroconvulsive treatment (ECT), transcranial magnetic stimulation (TMS), and transcranial direct current stimulation (tDCS) have been widely used for MDD treatment (Rosa and Lisanby, 2012; Padberg et al., 2021). In the past decade, in addition, transcranial alternating current stimulation (tACS), with its unique clinical potential to specifically manipulate intra- and interarea neural oscillations, has been shown to alleviate emotional and cognitive symptoms in neuropsychiatric disorders such as obsessive-compulsive disorder (Grover et al., 2021), schizophrenia (Farcas and Iftene, 2022), and Alzheimer’s disease (Benussi et al., 2022). Several latest reviews have claimed that tACS has been considered to have good potential for various clinical interventions (Elyamany et al., 2021; Grover et al., 2023) and it has also attracted increasing attention for MDD treatment. In this review, we first introduce major advances in tACS techniques, which greatly extend their application potential. Then, we summarize the underlying modulation mechanisms of tACS for treating MDD, from both local and interarea brain oscillation abnormalities in MDD patients. These findings provide modulation targets for tACS. Furthermore, we review the latest clinical studies on tACS applications in MDD and discuss the alleviation effects on MDD symptoms. Finally, we propose future directions to maximize the clinical value of tACS for MDD treatment.

## Advances in tACS methods

**FIGURE 1:**
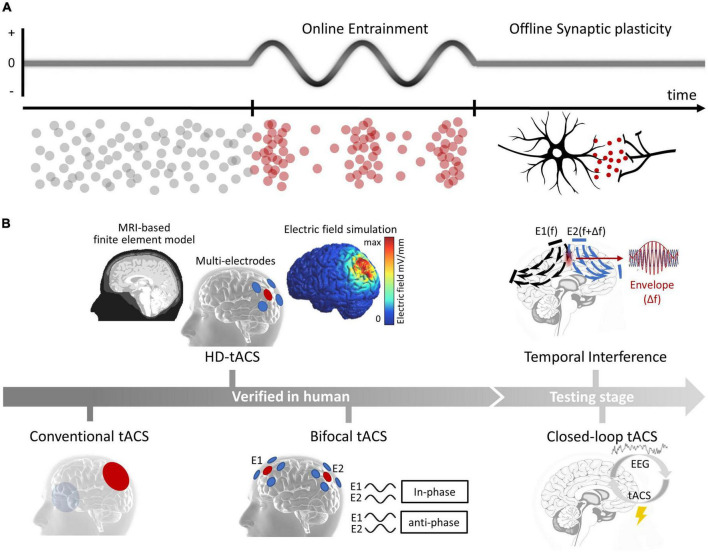
**(A)** Physiological mechanisms of tACS. Online entrainment effect and offline synaptic plasticity. **(B)** Advances of tACS. From conventional tACS to novel techniques that verified in human participants (HD-tACS and bifocal tACS) and that during early testing stages (temporal interference and closed-loop tACS).

Transcranial alternating current stimulation applies a weak, frequency-specific sine-wave alternating current to the cortex and modulates brain oscillations by entraining the intrinsic brain oscillations to the applied current, resulting in the phase alignment with external current and the increased power of neural oscillations with frequencies close to the applied current (Frohlich and McCormick, 2010; Herrmann et al., 2013; Helfrich et al., 2014a,b; Krause et al., 2019; Johnson et al., 2020). In addition, tACS can also induce a sustained poststimulation effect as continued power changes of neural oscillations with frequency close to the stimulation current (Wischnewski et al., 2019; Fiene et al., 2020). This offline entrainment effect been successfully predicted by a synaptic change model based on spike timing-dependent plasticity (STDP) (Zaehle et al., 2010) and has been directly observed through electroencephalography (EEG) in humans (Neuling et al., 2013; Vossen et al., 2015; Kasten et al., 2016; Figure 1A). Besides, some studies have also reported indirect long-distance effects of tACS. For instance, some studies have suggested the tACS on visual areas could evoke neural activity changes in retina and led to phosphene (Schwiedrzik, 2009); in an opposite direction, some studies found transcutaneous-ACS on peripheral nerves could modulated neural oscillations in central motor areas and affected behavioral performance (Zaghi et al., 2010; Asamoah et al., 2019). However, the mechanism underlying these long-distance modulations require more verifications in future studies.

The efficiency of conventional tACS, which uses two large electrodes immersed in saline-soaked sponges and guides their placements by the international 10–20 EEG system, was largely limited due to the poor current focus. Encouragingly, some recent advances have significantly improved the current focality of tACS (Figure 1B, left). One of the major improvements is high-definition transcranial electrical stimulation (HD-tES) technology, which uses an array of small disc electrodes arranged in a ring configuration. These array settings have been shown to significantly improve the stimulation focality (Datta et al., 2009; Dmochowski et al., 2011), and has been applied to modulate nearby brain subregions [such as the dorsolateral prefrontal cortex (DLPFC) and ventrolateral prefrontal cortex (VLPFC)] and could produce larger and more enduring behavioral and EEG effects (Kuo et al., 2013). Additionally, researchers have combined individualized brain structure data to create realistic finite-element models (FEM), which can guide a more precise placement of small disc electrodes (Datta et al., 2009; Salvador et al., 2010; Neuling et al., 2012). One latest study provided direct evidence that FEM-guided HD-tACS of the bilateral frontoparietal cortex is more effective in changing craving behavior than conventional tACS (Soleimani et al., 2022). Another important advance is bifocal tACS technique, which aims to modulate interarea functional connectivity. Studies have found that in-phase stimulation synchronizes neural activities between two brain areas, while anti-phase stimulation desynchronizes them; synchronization was estimated by parameters such as the phase-locked value, phase-amplitude coupling in EEG studies (Polania et al., 2012; Reinhart and Nguyen, 2019; Schwab et al., 2019; Hu et al., 2022) and functional connectivity measurements indexed by blood oxygenation dependent (BOLD) signal correlations in functional magnetic resonance imaging (fMRI) studies (Violante et al., 2017). Bifocal tACS has been used to modulate cognitive processes in healthy populations. For instance, in-phase theta tACS has been used to increase frontal–temporal synchronization, resulting in a significant enhancement in working memory capacity in older adults. More importantly, the enhancement effect was larger in the bifocal tACS condition than that in tACS of either the frontal or temporal site (Reinhart and Nguyen, 2019). HD-tACS and bifocal tACS, have substantially enhanced the efficacy to regulate abnormal brain oscillations and interarea functional connectivity, highlighting their potential use in clinical practice.

In addition, two other important technological developments are currently in the testing stages (Figure 1B, right). One is temporal interference (TI), which can activate neurons in deep brain areas through the interference of two high-frequency electrical signals without causing any change in neural oscillations along the current pathways (Grossman et al., 2017). A recent modeling study suggested that TI can affect the human hippocampus (Lee et al., 2020), and an investing study claimed that TI could be safe and well tolerated in human participants (Piao et al., 2022). The other development is closed-loop tACS, which has significant potential for treating neurological and psychiatric disorders with irregular patterns (Chen et al., 2023). Recent studies have indicated that the effect of tACS is influenced by the state of the ongoing brain oscillations (Krause et al., 2022). However, currently, closed-loop tACS has only been used in limited situations. For example, some studies have used closed-loop tACS during sleep to modulate memory processes (Lustenberger et al., 2016; Ketz et al., 2018). And real-time closed-loop tACS in awake participants requires more future tests.

Regarding the side effects of tACS, some mild symptoms such as temporal nausea, tinnitus, and dizziness have sometimes been reported during the stimulation (Bhattacharya et al., 2022; Wang et al., 2022), and high-intensity stimulation does in some particular functional areas may have a chance to evoke sensory hallucinations such as phosphene (Antal et al., 2008). However, fortunately, there are no salient adverse reactions reported in most tACS clinical research (even in the cases of mania and/or hypomania and clinical epilepsy diagnosis) (Matsumoto and Ugawa, 2017). Moreover, a recent case study has further suggested a good safety profile of tACS among pregnant patients (Wilkening et al., 2019). In sum, although the safety of tACS has been recognized, the stimulation settings should always be cautious given the individual differences in tACS tolerance.

## The neural mechanisms of tACS for treating MDD

**FIGURE 2:**
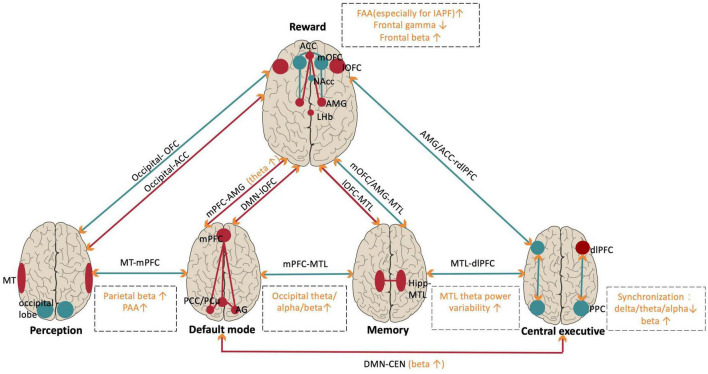
Abnormalities of local oscillations and oscillatory synchronization within and between relevant networks in MDD patients. Red indicates hyperactivation and hyperconnectivity. Turquoise indicates hypoactivation or hypoconnectivity in MDD. Orange indicates the brain oscillation abnormalities in specific frequencies within networks (in dashed square). Orange arrows indicate that brain oscillations support long-range functional connectivity. NAc, nucleus accumbent; mOFC/lOFC, medial/lateral orbitofrontal cortex; ACC, anterior cingulate cortex; AMG, amygdala; LHb, lateral habenula; MT, middle temporal; Hipp, hippocampus; MTL, medial temporal lobe; DMN, default mode network; PCu, precuneus; AG, angular gyrus; mPFC, medial prefrontal cortex; CEN, central executive network; dlPFC, dorsal lateral prefrontal cortex; PPC, posterior parietal cortex; FAA, frontal alpha asymmetry; IAPF, individual alpha peak frequency; PAA, parietal alpha asymmetry.

Accumulating studies have demonstrated both local oscillation abnormalities and interarea synchronization dysfunctions in MDD patients, and tACS may treat MDD by modulating such oscillatory abnormalities. To better understand the functional consequences of these abnormalities, we sorted out recent progress by functional networks, which could be responsible for different MDD symptoms (Figure 2). These findings provide candidate modulation targets for tACS intervention in MDD for clinical practice.

### Oscillation power abnormalities in the reward network

The human reward network mainly includes the prefrontal cortex and the limbic system (Dutcher and Creswell, 2018), and functional abnormalities in the reward network are closely associated with anhedonia, the core symptom of MDD (Pizzagalli and Roberts, 2022). Most MDD research has focused on the reward network and has demonstrated a wide range of local oscillation abnormalities.

Alpha band (8–12 Hz) power has been found to decrease with increasing brain activity (Goldman et al., 2002; Laufs et al., 2003). Researchers have consistently found that frontal alpha asymmetry (FAA), or the difference in alpha power between the right and left hemispheres, is closely related to MDD. Early studies found that FAA was significantly higher in depressed participants than in healthy participants (Henriques and Davidson, 1990), which is well explained by the approach–withdraw theory of emotion processing (Davidson, 1992); in this framework, the increased FAA in MDD patients reflects a decrease in approach motivation (supported by the left hemisphere) and an increased withdrawal motivation (supported by the right hemisphere). Besides, a recent study demonstrated that changes in FAA were closely associated with changes in the activity of the amygdala as well as the functional connectivity between the amygdala and the DLPFC, implying the increased FAA in MDD patients could also reflect increased negative emotional processing (Zotev et al., 2016). Currently, abnormal FAA in MDD has been replicated in a series of subsequent studies (Thibodeau et al., 2006; Grimm et al., 2008; Gollan et al., 2014). Moreover, accumulating intervention studies have provided evidence of a causal relationship between FAA and MDD. For example, targeting to decrease the FAA, studies adapted neurofeedback training (Peeters et al., 2014; Mirski et al., 2015; Wang et al., 2016), tDCS (Mirski et al., 2015; Al-Kaysi et al., 2017; Carvalho et al., 2020), and repetitive TMS (rTMS) (Olejarczyk et al., 2021) have found symptoms alleviations in MDD patients. Regarding the abnormal FAA in MDD patients, some recent studies explored whether it could predict the response to medication treatments but found mixed results (Baskaran et al., 2018), and a meta-analysis suggested that individual differences (such as sex and age), clinical characteristics (such as medication and comorbidities), and specific measurements (such as electrode selection and normalization methods) affected FAA in MDD patients (Van Der Vinne et al., 2017), indicating that it is essential to consider more factors when discussing abnormal FAA in MDD patients.

Gamma band (30–100 Hz) power is closely linked to increased local neuronal activities (Traub et al., 1998), and accumulating evidence suggests that MDD patients exhibit decreased gamma power during both the resting state and during the emotion-related tasks, particularly in the frontal lobe which is responsible for emotion regulation (Fitzgerald and Watson, 2018). There is also evidence further supports the close relationship between reduced gamma power and MDD. For example, MDD patients treated with ketamine showed significantly increased gamma power in a wide range of brain regions (Nugent et al., 2019). rTMS studies also found that stimulation of the left DLPFC elevated the gamma power in the resting state and that the increase in gamma power predicted improvement in depressive symptoms (Pathak et al., 2016; Noda et al., 2017). Nevertheless, some animal studies have found that gamma power reflects a combination of excitation and inhibition; thus, there may be an optimal gamma power that supports normal brain functions (Hajos et al., 2003; Mendez et al., 2012; Akhmetshina et al., 2016). However, there is no human evidence in support of this view yet.

Abnormalities in theta band (4–7 Hz) power in MDD have also been reported. Early studies found increased frontal–midline theta power in the resting state in MDD patients; this increase was located in the anterior cingulate cortex (ACC) and reflected impaired emotion regulation (Jaworska et al., 2012). However, some following studies did not replicate this finding and even found an opposite abnormal pattern (Jiang et al., 2016; Zhang Y. et al., 2022). Besides, some other studies have reported that the abnormal frontal theta asymmetry could be used as a biomarker to distinguish MDD patients from health populations (Kwon et al., 1996; Dharmadhikari et al., 2018), but its functional correlate is still unclear. Regarding theta power, two recent perspectives are worth noting. First, recent studies have suggested that abnormalities in theta band power are more related to anxiety than to depression (Gold et al., 2013; Zhang Y. et al., 2022); thus, the direct relationship between theta power abnormalities and MDD may require further studies to elucidate. Second, the interarea synchronization of theta power is critical for long-range information exchange (Reinhart and Nguyen, 2019), and recent studies have found that global theta synchronization is abnormally increased in individuals with depression compared to healthy individuals during the resting state and performance of emotion regulation tasks (Xing et al., 2017, 2019). We cover this latter point in detail when describing the abnormal interactions among brain networks.

In addition, beta band (14–30 Hz) abnormalities in MDD have also received increasing attention. Some studies using classifier methods reported that beta band features in the frontal lobe are the best at distinguishing MDD patients from healthy individuals (Cai et al., 2016; Shen et al., 2017). However, the causality between frontal beta oscillations and MDD symptoms has not been fully tested. Interestingly, recent studies consistently demonstrated abnormally increased parietal beta power in MDD patients. For example, one study found that parietal beta hyperactivity in MDD is correlated with the severity of anxiety among MDD patients (Grin-Yatsenko et al., 2010). More importantly, recent neurofeedback training studies have supported a causal relationship between parietal beta power and MDD by revealing alleviated anxiety and depressive symptoms after downregulating the parietal beta power (Wang et al., 2019), and the reduction in parietal beta power was correlated with an improvement in depressive symptoms (Chen and Lin, 2020). However, the functional role of parietal beta oscillation is still unclear.

To be note, a recent review has summarized the functional differences within the frontal–limbic reward network. For example, the ACC acts as a “control hub”; the medial orbitofrontal cortex (mOFC) and nucleus accumbens (NAc) act as “reward” hubs; and the lateral orbitofrontal cortex (lOFC), ventromedial prefrontal cortex (vmPFC), and amygdala act as “non-reward” hubs (Pizzagalli and Roberts, 2022). Besides, recent studies have identified that lateral habenula (LHb), as a pivotal node in the anti-reward circuit, also plays a crucial role in MDD (Liu et al., 2017; Liang et al., 2022; Zhang C. et al., 2022). However, limited by the inferior spatial resolution of regular EEG signals, it is challenging to accurately locate the sources of the oscillation power abnormities within the reward network.

### Oscillatory synchronization abnormalities among MDD-related networks

Recent findings have extended our understanding of oscillation power abnormalities in MDD beyond the reward network. Furthermore, researchers have demonstrated functional connectivity abnormalities among critical MDD-related networks and correlated them with different MDD symptoms (Kaiser et al., 2015; Rolls et al., 2018; Pizzagalli and Roberts, 2022). We summarized important findings from the synchronization perspective and discussed how they supported the functional connectivity findings.

The default mode network (DMN) consists of a group of brain regions that exhibit decreased activity during attention-oriented and goal-directed tasks, with primary hubs of the medial prefrontal cortex (MPFC), posterior cingulate cortex (PCC), precuneus (PCu), and angular gyrus (AG) (Raichle et al., 2001). Research on health populations has found that the anterior MPFC primarily involves self-referential psychological processes, whereas the posterior hubs are more closely related to recalling prior experiences (Raichle, 2015). A recent meta-analysis has indicated that abnormally increased activities in DMN is closely related to rumination in MDD patients (Ho et al., 2015; Kaiser et al., 2015) and this view is supported by abnormally reduced alpha power in the posterior parietal cortex (PCC) in MDD patients (Jaworska et al., 2012; Jiang et al., 2016). Furthermore, recent MDD research has identified abnormal strengthening of functional connectivity between the DMN and the reward network. For example, some studies have suggested that the abnormally increased functional connectivity among the MPFC, rostral ACC, and amygdala could be responsible for the increased interaction between self-referential processing and negative emotional information processing (Yamawaki et al., 2012; Kaiser et al., 2015). More importantly, the latest iEEG study on healthy individuals revealed that the communication between the MPFC and amygdala is mainly supported by theta oscillations (Chen et al., 2021).

The central executive network (CEN) plays a pivotal role in tasks requiring executive control, with key nodes including the DLPFC and posterior parietal cortex (PPC) (Sherman et al., 2014), and accumulating resting-state fMRI data indicates that decreased functional connectivity within the CEN hubs accounts for the typical cognitive deficits in MDD patients (Kaiser et al., 2015; Zhang et al., 2021). A recent EEG study examined synchronizations in broad frequency bands between the DLPFC and PPC and found that depressed patients exhibited lower task-dependent increases in the phase synchronization of the delta, theta, and alpha bands but a higher phase synchronization of the beta band (Li et al., 2017). Additionally, fMRI studies have found abnormal increases in activity synchronization between the CEN and the DMN in MDD patients, reflecting impaired inhibition of the DMN by the CEN (Lemogne et al., 2012). A following EEG study suggested such abnormally increased connectivity especially supported by beta power synchronization, which was observed in MDD patients during the acute phase but not the remission phase (Whitton et al., 2018).

In recent MDD research, considerable attention has been devoted to abnormalities in the perception system, particularly in the visual system. Earlier studies noted that individuals with MDD demonstrate a significant perceptual bias toward negative information (Leppänen, 2006) and an impairment in motor suppression (Golomb et al., 2009). The following studies have associated these perceptual dysfunctions with abnormally decreased gamma-aminobutyric acid (GABA) concentrations and increased neural activities in the occipital cortex and middle temporal area (MT) (Song et al., 2021; Liu et al., 2022). Additionally, recent fMRI studies found reduced connectivity of the occipital cortex/MT with the OFC but increased connectivity with the MPFC/ACC in MDD patients (Teng et al., 2018; Zhang et al., 2021), and some latest studies further proved the casualty between the latter abnormality with MDD symptoms by using magnetic stimulation (Zhang et al., 2021; Lu et al., 2022). However, except for an earlier EEG study which exhibited significantly increased resting-state theta, alpha, and beta power in the occipital lobe in MDD patients (Grin-Yatsenko et al., 2010), few synchronization abnormalities were reported within perception systems.

Besides, the hippocampal–medial temporal lobe (Hipp-MTL) network is known to play a pivotal role in human memory (Graham et al., 2010) and its dysfunction could account for the memory deficits in MDD patients. Accumulating studies have reported hippocampal atrophy (Sheline, 2011), abnormally increased metabolic activity in the hippocampus and parahippocampal gyrus (Kennedy et al., 2001; Videbech et al., 2002) and increased functional connectivity within the Hipp-MTL network in MDD patients (Rolls et al., 2018). From a neural oscillation perspective, recent research has emphasized the critical role of hippocampal theta power in anxiety regulation (Korotkova et al., 2018). However, except for an early MEG study that reported a correlation of the reduction in MTL theta power variability with the severity of depressive symptoms in MDD patients (Linkenkaer-Hansen et al., 2005), there is limited evidence linking reduced Hipp-MTL theta power to abnormal memory processing in MDD patients. Besides, some other studies have shown that functional connectivity abnormalities of the Hipp-MTL network with a wide range of brain areas. For example, some studies have found that functional connectivity of the Hipp-MTL network with the amygdala is reduced in depressed patients, and these reductions are positively correlated with the severity of depressive symptoms (Cheng et al., 2018; Rolls et al., 2018; He et al., 2019). Although a recent iEEG study suggested that phase-amplitude coupling between theta/alpha oscillations in the amygdala and high-frequency gamma activity in the hippocampus is critical for normal negative emotional processing (Zheng et al., 2017), no evidence for decreased synchronizations between hippocampus and amygdala in MDD patients has been reported yet. Some other studies have demonstrated abnormally reduced functional connectivity between the Hipp-MTL network and mOFC (Cheng et al., 2016; Rolls et al., 2020) but abnormally increased connectivity between Hipp-MTL network and lOFC (Rolls, 2016). However, the underlying oscillatory synchronization mechanisms are unclear yet.

### Oscillation frequency abnormalities

In addition to oscillation power and interarea synchronization, recent studies have examined abnormal brain activity in MDD from a novel perspective of oscillation frequency and have reported important findings regarding individual alpha peak frequency (IAPF). Studies on healthy populations have found that higher IAPF is usually associated with better cognitive ability (Scally et al., 2018) and has been found to decrease in a variety of mental illnesses associated with cognitive impairment (Garces et al., 2013; Dickinson et al., 2018; Ramsay et al., 2021). In recent years, more studies have explored the relationship between IAPF and depressive symptoms. For example, a study on healthy individuals found that IAPF positively predicted self-reported depression scores (Tement et al., 2016). Another recent study replicated this result, and they also found that the power of IAPF was negatively correlated with the self-reported depression score (Zhou et al., 2023). Although few studies have directly examined abnormal IAPF in depression patients, some studies have found that MDD-related alpha power (a.k.a. FAA), obtained based on IAPF, can better distinguish MDD patients from healthy individuals than that based on the standard alpha frequency band (8–12 Hz) (Gollan et al., 2014). In addition, a series of studies have found that IAPF can predict the efficacy of rTMS treatment for MDD. For example, some studies have found that the baseline IAPF is significantly higher in the rTMS-responsive group than in the rTMS non-responsive group (Arns et al., 2012) and that higher IAPF is correlated with greater alleviation of depressive symptoms after rTMS treatment (Corlier et al., 2019). However, the latest research suggests that IAPF may have a complex non-linear relationship with the effects of rTMS treatment, with the stimulation effect largest when the individual’s IAPF is closest to the stimulation frequency (Roelofs et al., 2021). Although there is no direct evidence of tACS treatment for MDD, previous studies have demonstrated that IAPF-based tACS intervention can improve human cognitive function in healthy populations (Zhang et al., 2019). Taken together, these results suggest the importance of considering individualized oscillation frequency for tACS applications in MDD interventions.

## Clinical studies on tACS applications in MDD

**FIGURE 3:**
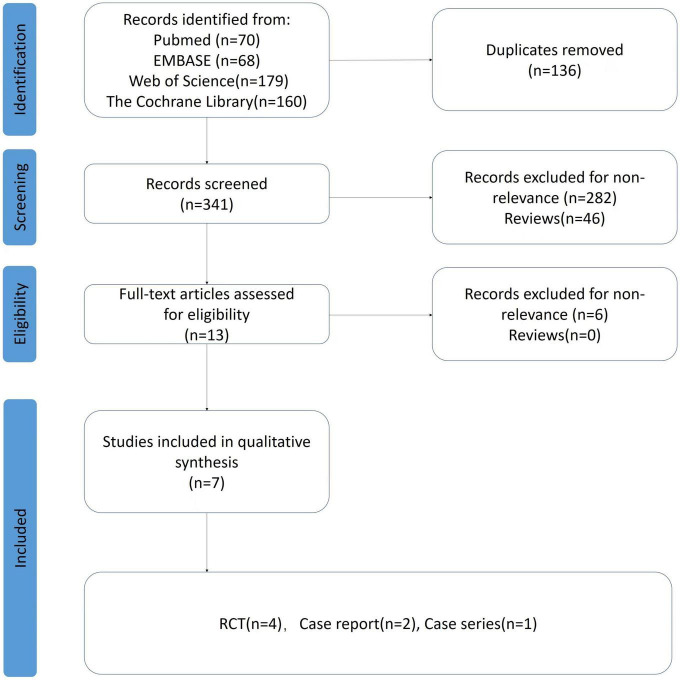
PRISMA flow chart for the search and inclusion criteria for tACS studies in MDD.

As the quick developments in tACS techniques and extended understanding of the brain oscillation abnormities in MDD, using tACS to treat MDD has attracted increasing attention in recent years. We conducted a systematic search in a wide range of English databases, including PubMed, EMBASE, Web of Science, the Cochrane Library, Register of Controlled Trials and the Chinese database Zhiwang; we used the following search terms: (“depress*” OR “major depressive disorder” OR “MDD”) AND (“transcranial alternating current stimulation” OR “tACS”). Initially, we selected articles based on their titles and abstracts and excluded duplicate articles. Then, we assessed the abstracts and full texts of potentially eligible studies according to predefined inclusion criteria. Finally, we included a total of seven published articles (see details in Table 1 and Figure 3).

**TABLE 1:** Clinical studies on tACS applications in MDD.

References	Study design	Participants	Stimulation protocol					Outcomes
			Session’s number	Session duration	Current amplitude (peak-to-peak)	Frequency	Electrode location	
Alexander et al., 2019	RCT	32 patients with unipolar depression without psychotic symptoms	Once daily for 5 consecutive days	40 min	2 mA at Cz 1 mA at F3 and F4	Group 1: 10 hz Group 2: 40 hz Group 3: sham	DLPFC: two electrodes over F3 and F4, and a third over Cz	1. No significant improvements in any group in MADRS at week 4 follow-up; 2. The 10 Hz group had more responders compared with 40 Hz and sham groups at 2 weeks after intervention; 3. A significant reduction in alpha power over the left frontal regions in 10 Hz group at day 5.
Riddle et al., 2020	Case report	3 patients with unipolar depression without psychotic symptoms	12 weekly sessions	40 min	2 mA at Cz 1 mA at F3 and F4	Patient 1: 10 hz Patient 2: 40 hz Patient 3: sham	DLPFC: two electrodes over F3 and F4, and a third over Cz	The patient received 10 Hz stimulation obtained remission (MADRS = 7) after 12 weeks
Wilkening et al., 2019	Case report	One pregnant MDD patient	Once daily for 9 consecutive days	20 min	2 mA	40 Hz	Bilateral DLPFC (F3 and F4)	1. The HAMD-21 and BDI scores decreased; 2. Cognitive test score (TMT) improved; 3. These effects sustained at 2 weeks and 3 months
Haller et al., 2020	Case series	6 MDD patients	Group 1: twice daily for 10 days Group 2: once daily for 10 days	Group 1: 10 min Group 2: 20 min	2 mA	40 hz	Bilateral DLPFC (F3 and F4)	1. HAMD-21 and BDI scores decreased 85.2 and 78.8% in Group 1, and 62.4 and 24.6% in Group 2; 2. Verbal n-back test and TMT performance improved
Wang et al., 2020	RCT	30 first-episode drug-naive unipolar MDD without psychotic symptoms	Once daily on weekdays for 4 consecutive weeks	40 min	15 mA	Group 1: 77.5 Hz Group 2: sham	PFC (Fpz, Fp1, and Fp2) Reference: bilateral mastoid	1. The response rate (HAMD-17 decreased ≥50%) of Group 1 was significantly higher than that of Group 2 at the 8th weeks 2. The remission rate (HAMD-17 ≤7) of Group 1 was significantly higher than that of Group 2 at the 4th weeks and 8th weeks; 3. No serious side effects, mania or hypomania
Wang et al., 2022	RCT	100 first-episode drug-naive unipolar MDD without psychotic symptoms	Once daily on weekdays for 4 consecutive weeks	40 min	15 mA	Group 1: 77.5 Hz Group 2: sham	PFC (Fpz, Fp1, and Fp2) Reference: bilateral mastoid	1. A greater reduction in depressive symptoms at 4th and 8th weeks; 2. No serious adverse events
McAleer et al., 2023	RCT	33 patients with depression and/or anxiety	2 verum sessions and 2 sham sessions	20 min	2 mA	Group 1: 6 Hz with a 180° phase difference Group 2: tDCS	tDCS: left DLPFC (F3); Fp2 tACS: two electrodes with highest synchronization among the whole brain	1. Reduction in anxiety, depression, and valence and arousal ratings in both groups, larger effects in tACS group; 2. In tACS group, little-to-no change in patients who received sham stimulation first, while significant improvements in patients who received verum stimulation first

RCT, randomized controlled trial; DLPFC, dorsolateral prefrontal cortex; HAMD, trail making test, Hamilton depression; BDI, Scale Beck Depression Inventory; MADRS, Montgomery-Asberg Depression Rating Scale; MDD, major depressive disorder; tACS, transcranial alternating current stimulation; tDCS, transcranial direct current stimulation.

### tACS to decrease frontal alpha asymmetry

Targeting the abnormally increased FAA in MDD patients, some clinical studies used 10-Hz tACS to balance bilateral DLPFC activities in MDD patients. A randomized controlled trial (RCT) estimated the frontal tACS effects on MDD among three groups receiving 10 Hz, 40 Hz, and sham stimulation (1 mA at F3/F4 and 2 mA at Cz, 40 min per day for 5 days). The bilateral 10-Hz tACS group showed a significant decrease in alpha power in the left frontal and central areas, resulting in decreased FAA among frontal areas. Although no significant alleviation of MDD symptoms was observed in any group immediately after the intervention, the response rate [defined as a decrease in Montgomery-Asberg Depression Rating Scale (MADRS) score ≥50%] was significantly higher in the 10-Hz tACS group than in the 40-Hz tACS or sham groups 2 weeks later (Alexander et al., 2019). Furthermore, a follow-up case study tracked three patients who received 10 Hz, 40 Hz or sham tACS weekly for 12 weeks. The results demonstrated that only the patient receiving the 10-Hz stimulation achieved clinical remission (MADRS score ≤7), and this effect lasted for 2 months (Riddle et al., 2020). These exploratory studies provide preliminary evidence of the efficacy of decreasing FAA for treating MDD.

### tACS to increase frontal gamma oscillation

Targeting the abnormal decreased gamma oscillations in MDD patients, latest clinical studies have also explored the modulation effects of prefrontal gamma tACS on MDD. Some pioneering case studies revealed the positive modulation effects of 40 Hz. For example, in a recent case study, the bilateral DLPFC (F3/F4) was targeted with 40-Hz tACS at 2 mA for 20 min per day in a pregnant woman with MDD. After receiving nine stimulation sessions within 2 weeks, the depressive symptoms [as assessed with the HAMD, Beck Depression Inventory (BDI)] decreased, and cognitive performance [as assessed with the Trail-Making Test (TMT)] improved. These effects were sustained at 2-week and 3-month follow-up assessments (Wilkening et al., 2019). Subsequently, Haller et al. (2020) investigated how different gamma tACS protocols influenced treatment efficacy. In this study, six patients were randomly divided into two groups, with one group receiving 10 min of stimulation twice a day and the other group receiving 20 min of stimulation once a day, with both groups receiving 10 stimulation sessions within 2 weeks. The stimulus parameters were 2 mA applied to the bilateral PFC (F3/F4) at 40 Hz. After 2 weeks of intervention, MDD symptoms (as assessed with HAMD and BDI) were alleviated and cognitive tests (n-back and trail making test) improved in both groups. In particular, the improvements were more significant in the group that received twice-daily stimulation. Moreover, some RCTs have focused on the tACS modulation effects of frontal gamma bands at higher frequencies on MDD. In a recent RCT, 30 first-episode, drug-naive MDD patients were randomly assigned to either the treatment or sham stimulation groups. One electrode was placed on the forehead (covering Fp1, Fpz, and Fp2), and two reference montages were placed on the bilateral mastoids. These three electrodes emitted a 15-mA alternating current at 77.5 Hz. The stimulation was administered for 40 min per day, 5 days per week and for 4 consecutive weeks. The results revealed that the depression severity scores (as assessed with the HAMD) in the treatment group were significantly lower than those in the sham group, and this effect persisted at the follow-up assessment 4 weeks later (Wang et al., 2020). Previous studies have shown that 77.5-Hz tACS increases the concentrations of beta-endorphin and serotonin in the cerebrospinal fluid, hypothalamus and cortex, producing an antidepressant effect (Lebedev et al., 2002; Zaghi et al., 2010). In a subsequent RCT study with a larger sample size (*n* = 100), the same tACS parameters were used. The results showed that there was a significant reduction in depressive symptoms and a higher response rate in the treatment group in both the immediate test and another test administered 4 weeks after the intervention. At week 4, the response rate (a decrease in HAMD-17 score ≥50%) and remission rate (HAMD-17 score ≤7) were higher than those in the sham group, and the difference in the response rate lasted until week 8 (Wang et al., 2022). Together, these studies indicate that multisession PFC gamma tACS helps to alleviate symptoms of MDD and improve cognitive function.

### tACS to reduce global theta synchronization

Targeting the abnormally increased theta synchronizations among the MDD-related networks, a recent RCT further tested if tACS desynchronizing theta oscillations could alleviate MDD symptoms. The researchers assigned 33 volunteers with depression and/or anxiety to either the tACS or tDCS group. Each participant received two 20-min stimulation sessions and two 20-min sham-stimulation sessions in a randomized order. In the tDCS group, F3 and FP2 were selected as the anodal and cathodal sites with a current of 2 mA. In contrast, in the tACS group, the strength of theta synchronizations among the whole brain was first calculated, and then a 6-Hz anti-phase current was passed between the two electrodes with the highest theta synchronizations. Although anxiety and depressive symptoms were improved in both groups, the degree of depressive symptom alleviation was more significant in the tACS theta desynchronization group. Further analysis also showed that participants who received the treatment first showed larger improvements than those who received the sham stimulation first (McAleer et al., 2023). This result encourages future studies to treat MDD from the interarea network perspective.

In summary, clinical tACS studies have shown promising improvements in alleviating MDD symptoms. However, we should note that the underlying modulation mechanisms of these studies were various and resulted in different outcomes. For instance, only gamma tACS studies emphasized the enhancement of cognitive performance in MDD patients. Furthermore, while most clinical studies focused on modulating neural oscillations in the frontal cortex, there was a lack of consistency in tACS settings. Discrepancies in electrode placement, stimulated frequency, and dosages could account for the mixed modulation results across studies, particularly in high-frequency interventions. Besides, it is also crucial to conduct more high-quality RCTs that directly compare different categories and settings of tACS modulation effects.

## Challenges and future directions

**FIGURE 4:**
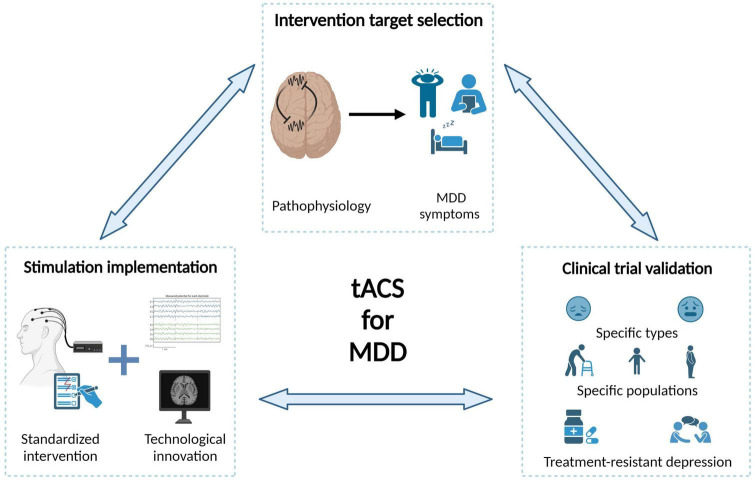
Challenges and future directions of tACS in the treatment of MDD. In-depth interdisciplinary collaboration is necessary to optimize the efficacy of each intervention step and fundamentally promote the clinical application of tACS for MDD treatment.

Although the significant progress in tACS technology, understanding of neural oscillations, and clinical practices in past decades, there are some challenges to the clinical translation of tACS. We suggest that in-depth interdisciplinary collaboration is necessary to optimize the efficacy of each intervention step and fundamentally promote the clinical application of tACS for MDD treatment. We summarize from three aspects: intervention target selection, stimulation implementation, and clinical validation (Figure 4).

### Intervention target selection

Selecting the targets for tACS intervention is fundamental to the success of intervention. Below, we summarize several challenges when choosing intervention candidates with the largest clinical values. First, the causal relationship between most neural oscillation abnormalities and MDD symptoms is unclear. We suggest that, at least three criteria should be satisfied for a tACS intervention candidate as follows: the neural oscillation differs in MDD patients compared to healthy individuals, the neural oscillation strength predicts the severity of MDD symptoms, and the neural oscillation strength returns to normal levels after treatment in case studies. Future studies should more focused on intervention evidence, especially for brain areas besides frontal cortex. Second, the relationship between neural oscillation abnormalities and information processing impairments should be further clarified. On the one hand, MDD studies have found that some neural oscillations exhibit opposite abnormal patterns between the resting state and functional states, suggesting that the functions of neural oscillations could be state-dependent. Therefore, future tACS interventions should consider neural states. On the other hand, the latest data-driven findings have revealed a series of neural features that can distinguish MDD patients from healthy individuals, decoding the mechanism of these abnormal neural features may greatly enlarge our pool of intervention candidates. Third, the correspondence between different symptoms of MDD and specific brain network abnormalities should be further explored. Researchers have summarized three main symptomatologic features of MDD (Malhi and Mann, 2018). Current research has mainly focused on mood and cognitive symptoms, with relatively few studies on neurovegetative symptoms. Additional research on sleep abnormalities in depressive populations is needed and investigating non-invasive interventions for MDD during sleep is an important direction for future studies.

### tACS implementation

The efficacy of tACS interventions cannot be guaranteed without a precise implementation procedure. Here, we summarize three main challenges for tACS implementation. Standardizing tACS intervention is the top priority. One of the most important aspects of an intervention protocol is determining key parameters, including stimulation sites, current intensities and frequency bands. To guarantee the implementation of a standardized procedure, it is also necessary to provide an operation manual as well as training for potential users. Another important aspect for developing a tACS treatment plan is knowledge of the effects of the number of stimulation sessions and intersession intervals, which should be explored more systematically. Moreover, overcoming critical technical challenges is essential for further improving tACS implementations, which requires more involvements of biomedical engineers. For example, it remains unclear how to effectively suppress (instead of increase) brain oscillations in a specific frequency (e.g., inhibit the hyperactivity of parietal beta oscillations), and how to modulate an abnormal oscillation pattern that consists of more than one targeted frequency or consists of signals from multiple brain areas (e.g., feature combinations from classifier findings). The advances in tACS intervention techniques can better meet various clinical needs for MDD treatment. Another challenge is the development of tACS technology that is compatible with non-invasive neural imaging techniques. Currently, direct measurements of the current distribution of tACS mainly obtained through invasive electrophysiological recordings, and for most application cases, the electrical current distribution of tACS can be determined only by simulations. Solving this technical problem would enable direct observation of the immediate and subsequent effects of tACS intervention and help us better understand its underlying mechanism, providing a gold standard to evaluate intervention efficacy.

### Clinical validation

Clinical validation is vital in determining whether a specific tACS intervention is suitable for widespread use in MDD treatment, and there are several significant challenges in current clinical practice. First, the evidence for the effects of different tACS frequencies on psychiatric symptoms is inconclusive, and clear neurophysiological mechanisms are lacking. The immediate priority is conducting more confirmatory RCTs for several promising tACS intervention plans. The clinical value of tACS should be further emphasized to potential users such as psychiatrists. Second, the highly complex and dynamic nature of MDD symptoms, which require symptom-dependent intervention plans. Depression is highly comorbid with other psychiatric disorders, especially anxiety (Tiller, 2012). Additionally, a significant proportion of unipolar MDD patients may develop bipolar disorder during treatment (Ratheesh et al., 2017). Some recent studies have found that neural oscillation abnormalities reported in MDD studies may be more closely related to anxiety, and the neural states during unipolar and bipolar periods could be greatly different. Therefore, tACS intervention plans should be developed not only based on patients’ current state but also their potential future states. Third, the interaction between tACS and other treatment methods must be fully considered in future clinical practice. As the first-line intervention for MDD, drug treatment has been found to impact the efficacy of TMS or tDCS interventions (Connolly et al., 2012; Wang et al., 2021), but few studies have examined such influences on tACS interventions. For example, previous studies have found that ketamine primarily regulates gamma oscillations among multiple brain areas (Nugent et al., 2019). Does this mean that tACS targeting gamma oscillations should not be recommended for patients receiving ketamine to avoid any interference, or will tACS result in a better integration effect? Similarly, psychological treatments, such as cognitive–behavioral therapy, interpersonal psychotherapy, and mindfulness therapy, have also been widely used in MDD treatment, and future research should aim to improve intervention efficacy by combining the strengths of each type of intervention.

## Conclusion

In the past decade, tACS has demonstrated unique clinical potential in precisely modulating both local and interarea abnormal brain rhythms. Accordingly, accumulating studies have significantly advanced our understanding of brain oscillation abnormalities in MDD from these two perspectives, providing a series of tACS intervention options for MDD treatment. Moreover, pioneering clinical trials have shown preliminary treatment effects of tACS, indicating its potential as a viable MDD treatment option. However, to facilitate the potential clinical application of tACS, further causality studies on neural abnormalities and MDD, research on standardized tACS intervention procedures, and additional RCTs are needed.

## Author contributions

YC and QC flameworked this review, wrote the future direction session, and generated the final manuscript. YZ, SY, and RP wrote the preliminary manuscript for the advances of tACS, neural abnormalities in MDD, and clinical studies. All authors contributed to the article and approved the submitted version.

## Footnotes

1
https://www.who.int/news-room/fact-sheets/detail/depression
